# Autophagy and cancer stem cells: molecular mechanisms and therapeutic applications

**DOI:** 10.1038/s41418-019-0292-y

**Published:** 2019-02-06

**Authors:** Francesca Nazio, Matteo Bordi, Valentina Cianfanelli, Franco Locatelli, Francesco Cecconi

**Affiliations:** 1 Department of Oncohaematology and Cellular and Gene Therapy, IRCSS Bambino Gesù Children’s Hospital, 00165 Rome, Italy; 2 Department of Biology, University of Tor Vergata, 00133 Rome, Italy; 3 Cell Stress and Survival Unit, Center for Autophagy, Recycling and Disease (CARD), Danish Cancer Society Research Center, 2100 Copenhagen, Denmark; 4 Department of Gynecology/Obstetrics and Pediatrics, Sapienza University of Rome, Rome, Italy

**Keywords:** Macroautophagy, Cancer stem cells

## Abstract

Autophagy and mitophagy act in cancer as bimodal processes, whose differential functions strictly depend on cancer ontogenesis, progression, and type. For instance, they can act to promote cancer progression by helping cancer cells survive stress or, instead, when mutated or abnormal, to induce carcinogenesis by influencing cell signaling or promoting intracellular toxicity. For this reason, the study of autophagy in cancer is the main focus of many researchers and several clinical trials are already ongoing to manipulate autophagy and by this way determine the outcome of disease therapy. Since the establishment of the cancer stem cell (CSC) theory and the discovery of CSCs in individual cancer types, autophagy and mitophagy have been proposed as key mechanisms in their homeostasis, dismissal or spread, even though we still miss a comprehensive view of how and by which regulatory molecules these two processes drive cell fate. In this review, we will dive into the deep water of autophagy, mitophagy, and CSCs and offer novel viewpoints on possible therapeutic strategies, based on the modulation of these degradative systems.

## Abstract (web summary)

Autophagy and mitophagy are deregulated in many types of cancer stem cells (CSCs). Although there is yet to be discovered, both autophagy and mitophagy are able to regulate different steps of CSCs physiology such as metabolism, stemness, migration and chemo-resistance.

## Graphical abstract

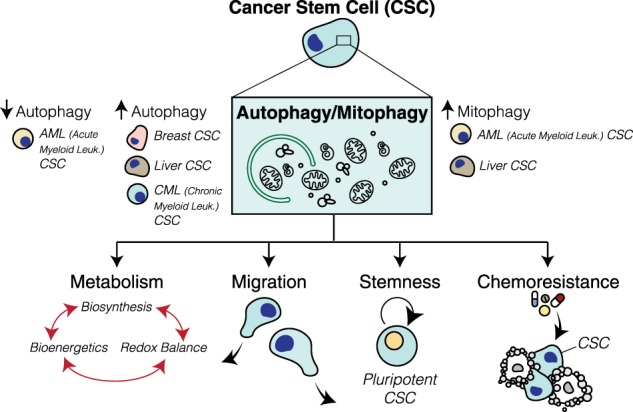

## Facts

- Autophagy plays a dual role in cancer as a tumor suppressor and promoter.
- CSCs are usually characterized by a dysregulation of autophagy/mitophagy.
- The modulation of autophagy/mitophagy impacts on CSC generation, differentiation, plasticity, migration/invasion and pharmacological, viral and immune-resistance.
- Targeting autophagy/mitophagy could pave the way for new therapeutic strategies to fight CSC aggressiveness.

## Open questions

- What are the key signaling pathways impacted by autophagy in CSCs?
- Which is the role of mitophagy in the profound metabolic reprogramming of CSCs?
- How could we manipulate autophagy to drive CSC fate?

## Autophagy in cancer: an overview

Autophagy is a self-digestion mechanism, in which cytoplasmic materials, proteins (in a pathway commonly defined as macroautophagy), damaged organelles, such as mitochondria (termed mitophagy), and lipids are sequestered into vesicles, called autophagosomes, for degradation and recycling. In basal conditions, autophagy is crucial for the preservation of cell homeostasis, acting as a protein/organelle quality control mechanism; during stressful conditions, such as starvation, hypoxia, and chemo/radiotherapy, it is instead fundamental for a cancer cell survival and adaptability to the perturbations of tumor microenvironment.

Autophagy has been initially attributed both tumor-suppressive and tumor-promoting functions. Opposite functions were interpreted as autophagy being a double-edged sword in cancer, and challenged researchers to further explore its impact on oncogenesis and tumor progression. Next, research in the field clearly demonstrated that the autophagy role in cancer exhibits a significant degree of context dependency, making us aware of the need to strictly relate each finding to its own and proper experimental system: e.g., the type/stage of tumor, the local (microenvironment)/systemic extracellular *milieu* of the tumor, the treatment with a specific cancer therapy or the genetic context.

Indeed, the accelerated oncogenesis observed in murine models defective for autophagy strongly supports the notion that autophagy prevents malignant transformation [1–3]. This tumor-suppressive function mostly occurs through the maintenance of the physiological tissue homeostasis, and empowers the pre- malignant cells to escape genotoxic stress and inflammation [4, 5], which both promote tumorigenesis. Such a cytoprotective role turns into a weapon serving cancer cells, and allowing them to cope with stress (metabolic, genotoxic, and inflammatory), which occurs after the malignant transformation is induced by anticancer therapy [5, 6]. Besides safeguarding cellular homeostasis, autophagy also affects cellular processes, such as epithelial-to-mesenchymal transition and migration, with both processes driving tumor progression and metastasization [7–9]. Altogether, autophagy can both promote and suppress cancer progression and metastasis at several stages. Notably, while autophagy induction is often a side effect of chemotherapy [10–12], it also has a beneficial role in cancer therapies involving induction of immunogenic cell death [13]. Hence, in order to exploit autophagy activation/inhibition for cancer treatment, it would be crucial to carefully assess the dependence/sensitivity of each specific type of cancer to autophagy, as well as the impact of autophagy modulation on selected cancer therapies.

## The cancer stem cell models

Cancer stem cells (CSCs, also known as tumor-initiating cells or tumor-propagating cells) are a small subpopulation of cancer cells that are responsible for tumor heterogeneity, displaying high metastatic potential and resistance to conventional anticancer therapy [14]. CSCs have been first identified in acute myeloid leukemia [15, 16] and then in many solid cancers, such as breast, pancreatic [17, 18], colon [19, 20], melanoma [21, 22], ovarian [23] and lung [24], and brain cancers [25, 26]. They are immortal tumor-cells that possess extraordinary self-renewal and differentiation capabilities that give rise to different phenotypes. CSCs are defined by the expression of specific cell surface markers that can be used to distinguish them from other tumor or normal cells. This opened the way to establish many in vitro and in vivo strategies to isolate and manipulate CSCs. Another important feature defining CSCs is the ability to recapitulate the original malignancy when transplanted in immune-deficient mice [14]. Breast cancer was the first human solid tumor proven to consist of heterogeneous populations of cells: non-CSCs and CSCs; specifically the CSCs subpopulation (CD44^+ ^CD24^−^/low) is capable of initiating tumor growth in immune-deficient mice [27]. Besides the capability of these cells to self-renew, accumulated evidence has established that a stronger resistance than non-CSC populations to anticancer therapies characterizes them. The failure of conventional treatments is strictly related to the plasticity of CSCs that, owing to their (1) deregulated self-regeneration and differentiation proprieties, (2) proliferative potential, (3) capability to be a quiescent cell pool, are most likely responsible for tumor initiation, progression, recurrence, and invasion. Overall, the identification of molecular mechanisms implicated in CSC survival remains crucial for augmenting the efficacy of presently available treatment regimens.

At least two main different models have been proposed to account for tumor origin and heterogeneity: the stochastic model and the hierarchical model. According to the first one, all cancer cells have the capability to give rise to new tumors by converting non-CSCs to a CSC phenotype in a dynamic way and in response to specific stimuli. By contrast, the hierarchical model is based on the concept that a unique population of CSCs produces the tumor and gives rise to heterogeneity by generating both differentiated and quiescent cancer cells. Although these models seem to exclude each other, what does happen is probably a combination of both things.

**Fig. 1:**
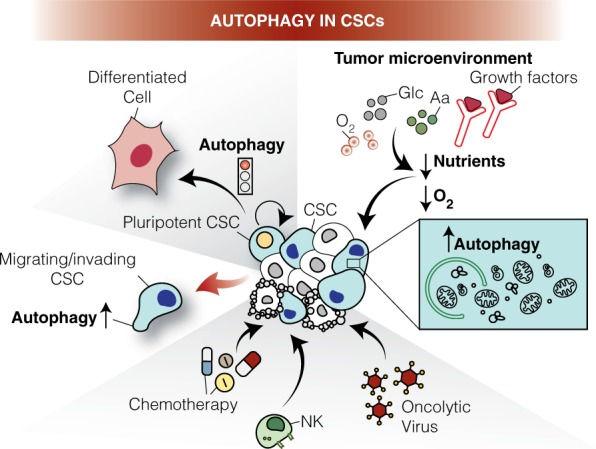
Roles of autophagy in cancer stem cells (CSCs). Tumor cells are heterogeneous and include cancer stem cell (CSC) populations. CSCs are often characterized by high levels of autophagy that (1) maintain pluripotency; (2) cope with low nutrients and oxygen levels (hypoxia) in the tumor microenvironment; (3) regulate CSCs migration and invasion; (4) promote resistance to chemotherapy, (5) help to escape immunosurveillance; (6) support oncovirus capability to infect, replicate in and kill them. In this scenario, autophagy manipulation is found to be crucial for the effective targeting of cancer cells. Aa: Amino acids; Glc: Glucose; NK: Natural Killer; O_2_: Oxygen

One of the pivotal processes that have been strongly associated to CSCs maintenance and aggressiveness is *autophagy*. In this review, we describe the role of both autophagy and mitophagy in CSC biology and discuss how their targeting could interfere with CSC survival. We will thus dig into how autophagy/mitophagy act and contribute to each step of CSC physiology: generation, differentiation, plasticity, migration/invasion and pharmacological, viral and immune-resistance (Fig. 1; for a summary see Table 1).

**Table 1:** Autophagy signature in cancer stem cells (CSCs)

Cancer stem cells	Upregulated autophagy gene expression	Targeted genes and treatments	Function of targeting/treatment
Breast CSCs	ATG5-12 LC3B *(32)* BECLIN 1 *(31)*	mTOR inhibitor FIP200, ATG7, LC3 ATG4C and ATG12 KD CQ Salinomycin	Induction of metastatic resistance Decreased pluripotency, CSCs maintenance, migration Inhibition of tumor formation Reduction of breast CSCs number
Ovarian CSCs	ATG5 *(33)*	BafA1 or ATG5 KD	Reduction of self-renewal
Glioblastoma CSCs	BECLIN 1, LC3 *(34)*	ATG4B KD in combination with radiotherapy	Slowed tumor growth
Pancreatic CSCs	HIF-1a BECLIN 1 LC3B *(56)*	HIF-1-a KD 3-MA	Promotion of the dynamic equilibrium between CSCs and non-CSCs
Chronic myeloid leukemia (CML) CSCs	*(37–39)*	ATG5 and ATG7 KD CQ and Lys05	Increased TKI-induced cell death
Acute myeloid leukemia (AML) CSCs	FIS1 *(114)*	ATG7 and LC3B KD CQ or BafA1 FIS1 KD	Overcome hypoxia-induced resistance Attenuated mitophagy and impairment of self-renewal potential
Gastric CSCs	LC3B *(69)*	CQ in combination with 5-FU	Cell death
Hepatic CSCs	ATG5, ATG7, BECLIN 1 *(31)*	CQ ATG5 KD BafA1, 3-MA CCCP	Increased apoptosis and decreased clonogenic capacity of CD133+ Reduction of hepatic CSC populations

*CQ*chloroquine, *3-MA*3-methyladenine, *BafA1*bafilomycin A1, *5-FU*5-fluorouracil, *KD*knockdown

## Autophagy and cancer stem cells

### Autophagy in the maintenance and survival of CSCs

**Fig. 2:**
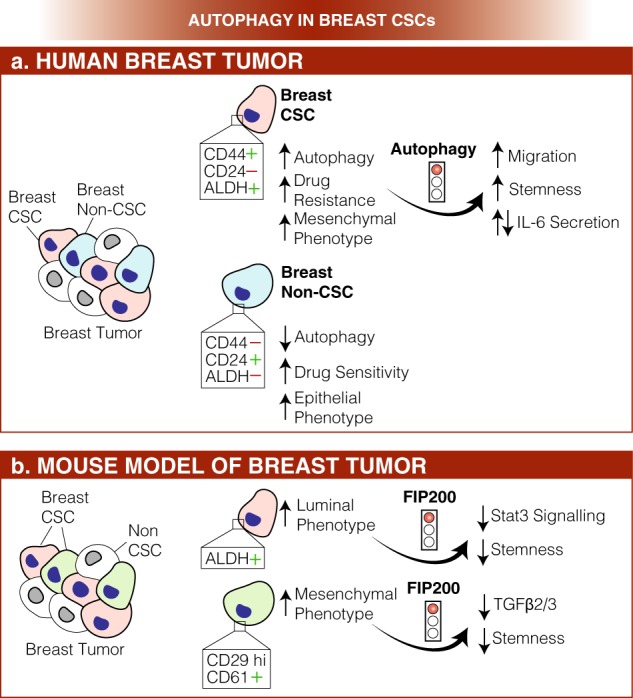
Role of autophagy in breast cancer stem cells (CSCs). **a** In human breast cancer, two different populations of cancer cells co-exist, the breast and non-breast CSCs. While the first population is characterized by high autophagy levels, increased resistance to chemotherapy and mesenchymal phenotype, the latter shows decreased autophagy, higher sensitivity to drugs and an epithelial, rather than mesenchymal, phenotype. Also, breast and non-breast CSCs differentially express cell surface markers (CD24, CD44, and ALDH). Importantly, autophagy inhibition results into enhanced migration and stemness and alteration in IL-6 secretion. **b**. In a mouse model of breast tumor, two different populations of breast CSCs have been isolated: a luminal one (in pink) and a mesenchymal one (in light blue). Intriguingly, all stemness markers (ALDH, CD29, and CD61) are downregulated in both populations upon FIP200 depletion, and this event correlates with Stat3 or TGFβ2/3 signaling downregulation, respectively. Also in this case, the two different populations of CSCs are distinguished by differential expression of cell surface markers (ALDH, CD29, and CD61)

In line with normal stem cells, CSCs are characterized by ability to self-renew and a *limited* differentiation capacity [14]. Pluripotency is a key feature of CSCs that allows them to indefinitely divide and maintain the undifferentiated state. By using fluorescence activated cell sorting (FACS) based on CD34 and CD38 (CD34^+ ^CD38^−^) surface marker expression, John Dick isolated the first CSCs from acute myeloid leukemia (AML) [15, 16]. By studying breast CSCs, it was made clear that autophagic homeostasis is an intrinsic feature for the maintenance of pluripotency under various pathophysiological conditions (Fig. 2a, for detailed description see ref. [28]). In fact, autophagy is upregulated in the mammospheres [29, 30] when compared to adherent cells, and both BECLIN 1 and ATG4, two key autophagy proteins, are needed for their maintenance and expansion. More recently, autophagy has been related to a variety of CSCs, such as breast [31, 32], pancreatic, liver [33], osteosarcoma [34], ovarian [35], and gliobastoma [36] CSCs, in which its impairment negatively affects the expression of staminal markers and consequently the cell self-renewal capacity. In hematological malignancies, depending on the context, autophagy can act both as a chemoresistance or tumor-suppressive mechanism. It is now clear that depending on both the type of progenitors and the state of leukemia disease (initiation versus progression), autophagy could have opposite roles. For example, in CML some autophagy-related genes (such as ATG4, ATG5, or BECLIN 1 [37–39]) are upregulated and the silencing of both ATG7 or ATG4B affects cell survival; so, the levels of autophagy in CML seems to be closer to solid tumor CSCs. On the contrary, functional autophagy is essential for protecting the evolution of myelodysplastic syndrome (MDS) to AML [40] and many autophagy-related genes are mutated or downregulated in some AML patients [41].

In recent years, researchers put a great deal of effort in understanding the molecular mechanisms of autophagy-dependent CSCs maintenance. Different signaling pathways have been identified: in MMTV-PyMT transgenic mice (a mouse model of breast CSCs), Yeo et al. [42] demonstrated that autophagy acts through EGFR/Stat3 and Tgfβ/Smad signaling in two distinct breast cancer stem-like cells (ALDH^+ ^and CD29hiCD61^+ ^, respectively, see Fig. 2b). Upon *FIP200* depletion, they found a decrease in the phosphorylation of EGFR, with this resulting in decreased STAT3 activation and consequently in an impairment of ALDH^+ ^breast cancer stem cells (BCSCs) tumorigenicity. On the other hand, autophagy inhibition leads to decreased TFGβ2 and TGFβ3 expression, inducing a defect in Smad signaling, which is indispensable for the CD29hiCD61+ CSCs phenotype. More recently, in the triple-negative type of autophagy-dependent BCSCs, it has been found that autophagy inhibition decreases the secretion of IL-6, probably through the STAT3/JAK2 pathway [43]. IL-6 secretion is crucial for CSC maintenance [44] and sufficient to induce the CD44^ + ^/CD24 low phenotype in breast cancer cell lines and tumors, this supporting the idea that the IL-6-JAK2-STAT3 signal transduction pathway could play an important role in the conversion of non-CSCs into CSCs.

Other studies suggest a role for FOXOs in regulating the fate of CSCs [45]. Although it is well described that FOXO-dependent regulation of transcription is fundamental to preserve homeostasis of stem cell in both embryos and adults [46], it still needs to be defined how FOXO activity could affect CSCs functions. Knockdown of FOXO3 results in increased CSC self-renewal capacity in prostate, glioblastoma, ovarian, breast, liver, and colorectal cancer [47–51]; in contrast leukemia-initiating cells need FOXO3 for stem cell maintenance [52, 53]. In the autophagy context, FOXOs have been reported to mediate the transcription of some autophagy-related genes (*ATG5, ATG8, ATG12, ATG14, BECLIN 1, ULK1, LC3, GABARAPL1, and BNIP3*, reviewed in ref. [54]) and as such cytosolic FOXOs participate in autophagy regulation. Very recently, the pro-autophagic protein AMBRA1 was found to be crucial for regulatory T-cell differentiation and homeostasis, acting on FOXO3-FOXOP3 axis. This opens new scenarios that may involve autophagy in FOXO3-mediated regulation of a lot of cellular processes [55].

However, further investigation is necessary to understand how FOXO-dependent regulation of stemness and autophagy are interconnected in tumorigenesis, considering that this is not simply a linear readout.

Intriguingly, recent findings suggest a crosstalk among autophagy, NAD^ + ^biosynthesis pathway and staminal markers; Sharif et al. [56] found that any perturbation in basal autophagy (generated by using both autophagy inhibitors and activators) decreases the pluripotency of teratocarcinoma CSCs, leading to differentiation and/or senescence.

Altogether, this evidence highlights the complexity of the autophagy-dependent regulation of CSCs.

Very recently, a novel link between autophagy and stemness arises from studies in ovarian cancer stem cells (OCSCs) [35]. Peng et al. found that Forkhead Box A2 (FOXA2) is overexpressed in ovarian CSCs and regulated by autophagy activity; inhibition of autophagy by both genetic and pharmacological approaches induces FOXA2 downregulation and, consequently, impairment of self-renewal ability.

Finally, other studies suggest a role  for autophagy in the regulation of chromosome stability by coordinating the ATR checkpoint and double-strand-break processing [57]. This opens the possibility that CSCs may exploit autophagy to prevent further DNA damage after an initial insult and maintain their survival.

### Autophagy as an adaptive mechanism of CSCs in the tumor microenvironment

Accumulating evidence indicate that the CSCs behavior is regulated by both extracellular signals (including hypoxic, metabolic, and oxidative stress) and intrinsic signals within CSCs that can promote self-renewal and plasticity. In particular, environmental stresses (such as lower oxygen levels, higher lactate levels, extracellular acidosis, and depletion of nutrients) are considered crucial for the maintenance of CSCs. It is suggested that stem cells lose the possibility for continued self-renewal when removed from their environment, the stem cell niche, which implies an essential role for microenvironment in directing stem cell fate [58]. It is well known that hypoxia commonly results in autophagy, mediated by the hypoxia- inducible factor 1alpha (HIF-1*α*). Hypoxia-induced autophagy has been demonstrated to be crucial for survival of liver CD133+ CSCs; more interestingly, in pancreatic CSCs, HIF-1*α*-dependent autophagy is critical for the equilibrium between non-stem pancreatic cancer cells and pancreatic CSCs [59]. Indeed, autophagy was found to be upregulated in multiple human AML cell lines and primary blasts after prolonged exposure to hypoxia; also, inhibition of the late-stage of autophagy overcomes Leukemia Stem Cells (LSCs) survival and chemoresistance [60, 61]. This highlights the controversial role of autophagy in the death/survival of leukemic cells, in which reduced autophagy appears to be an adaptive mechanism that accelerates AML development [62].

While advances have been made in understanding the role of autophagy and hypoxia in cancer, very little is known about the potential role of hypoxia-induced autophagy in maintaining the cancer stem cell niche.

### Autophagy role in migration/invasion of CSCs

EMT (epithelial-to-mesenchymal transition) is a critical event during embryonic development, determining changes in cell polarity and cell–cell contact. It is now clear that EMT signaling and CSCs phenotypes are strictly connected and a key feature of CSCs is their capability to migrate, which enhances their metastatic potential [27, 63, 64]. There is now increasing evidence that autophagy signaling and EMT are linked one to another [8, 65–67] and autophagy is often highly expressed in tumor cells carrying a mesenchymal signature [68]. In breast CSCs, autophagy inhibition (for instance, by ATG12 downregulation and chloroquine treatment) impairs the migratory and invasive cellular state, leading to increased expression of the epithelial marker CD24 and a decrease of vimentin (a mesenchymal cell marker) [69]. Moreover, in glioblastoma CSCs, two autophagy regulators, DRAM1 and SQSTM1 were found to be upregulated and to correlate with the expression of mesenchymal factors [67], thus  supporting the idea that autophagy controls migration/invasion in CSCs [10, 11]. In some solid tumors, such as glioblastoma, pancreatic, gastric and breast CSCs, autophagy negatively correlates with EMT; autophagy inhibition, indeed, is able to impair migration and invasion, while autophagy upregulation restores the mesenchymal phenotype. There are also evidences that EMT and autophagy could be considered two distinct processes, both correlated to the heterogeneous nature of CSCs (non-cycling and cycling CSCs) [68]. Accordingly, a hierarchical model defined the existence of cycling or non-cycling CSCs that come from EMT tumor cells: EMT tumor cells would be first induced to become autophagic CSCs (non-cycling) and, subsequently, cycling CSCs (featuring low autophagy). One possible hypothesis is that autophagic CSCs could induce EMT in other tumor cells upon release of EMT-inducing paracrine factors.

### Autophagy-mediated chemo and immune-resistance of CSCs

Despite advancements in radiation treatments and chemotherapy, which often target highly proliferating cells, it is clear that CSCs, living in a quiescent state and acquiring resistance to conventional therapy, are responsible for tumor recurrence. Different mechanisms through which CSCs can resist to drug-therapy have been proposed:

(1) Components of the CSC niche (immune cells, endothelial cells, fibroblasts and peri-vascular cells) protect CSCs from therapeutic interventions; (2) cellular plasticity; (3) high efficiency in repairing DNA damage; (4) high levels of MDR (multi-drug resistance) gene expression, or (5) inhibition of apoptosis. A relationship between CSCs and drug resistance has been found in many human cancers such as leukemia, melanoma, brain, breast, pancreatic, and colorectal cancers [70]. One of the model proposed is based on the existence of an intrinsic chemoresistance; this results in the persistence of a population of cancer cells that then leads to relapse following treatment. Among the mechanisms that have been proposed to confer resistance to chemotherapeutics, autophagy seems to be crucial. Moreover, it is well recognized that chemotherapeutic treatments are per se able to induce autophagy in cancer cells [71]. Several experimental approaches reveal that combining cytotoxic drugs and autophagy inhibitors increase CSCs sensitivity [72]. For example, in glioblastoma stem cells, Bevacizumab, a blocker of EGFR, or Temozolomide, in combination with chloroquine (a late-stage autophagy inhibitor), enhance drug toxicity, thus affecting glioblastoma CSCs survival [73, 74]. Very recently, Li et al. [75] found that in gastric CSCs the triple combination of 5-fluorouracil, chloroquine, and Notch inhibitor decreases cell viability and treatment resistance. Along similar lines, JAK-mediated autophagy was found responsible for preservation of stemness in cisplatin-resistant bladder cancer cells [76]; in agreement with this modulation, in glioma (GSCs) or AML CSCs, knockdown of ATG7 potentiates the inhibitory effect of salinomycin on cell survival [77]. In CML, Bellodi et al. [78] showed that the combination of autophagy inhibitors such as chloroquine or bafilomycin A1 (another late-stage autophagy inhibitor) with tyrosine kinase inhibitors (TKI) affects CML cell survival.

On the other hand, there are some evidence of the role that autophagy may play in drug-mediated cytotoxicity. In particular, resveratrol acts on breast CSC survival by inhibiting the Wnt pathway that, in turn, induces autophagy [79]; By contrast, the inactivation of mTOR stimulates neuroblastoma and glioma CSCs differentiation [80, 81]. In sum, uncovering the individual contribution of autophagy to CSCs drug resistance remains crucial for the development of novel antineoplastic therapies.

Besides the CSC resistance to chemotherapy, it is now emerging that CSCs are able to escape from the immune system through a process termed immunoresistance. Immune system is able to detect and interact with tumor cells through a mechanism named immunosurveillance. However, CSCs have been shown to be less immunogenic by both evading immune recognition and manipulating the immune system to stimulate their own growth; indeed, they achieve this by (1) producing immunosuppressive factors, (2) recruiting immunosuppressive cell types, (3) losing the expression of tumor antigens [82], through activation of distinctive cellular pathways such as Notch and Wnt. Moreover, CSCs produce cytokines to inhibit immune response: breast and glioblastoma CSCs are able to produce more TGFβ than normal cancer cells, and colon CSCs secrete IL-4 to inhibit antitumor immune responses. Of note, recent findings have revealed that autophagy contributes to immunosuppressive-related chemoresistance and promotes the capability of the tumor to avoid immune detection [83, 84]. Along the same line, enhanced autophagy has been observed in advanced stages of metastatic diseases, characterized by low levels of tumor-infiltrating T lymphocytes (TILs) [85]. Although this area of research is still poorly explored, autophagy could be considered a critical process for counteracting CSCs resistance to immunotherapeutic approaches.

### Autophagy and oncolytic virotherapy in CSCs

A new frontier in cancer treatment is represented by oncolytic viruses (OVs, including adenoviruses, herpes simplex virus, measles virus, reovirus, and Newcastle disease virus) that bypass the above-mentioned mechanisms of chemoresistance, thus efficiently killing CSCs in some cancer types [86]. OVs have the capability to infect, replicate in and kill cancer cells that express high levels of some virus-receptors, including CAR, CD46, or CD155. In the context of autophagy, there is accumulating evidence that, indeed, a role for OVs in the perturbation of cellular autophagy does exist [87]; on the other hand, the increased autophagy of CSCs could be exploited to target these cells by OVs. It has been proposed that a range of factors can promote or inhibit autophagy during the process of oncolytic adenoviral therapy, including infection, replication, and cell lysis [87]. During the replication step, adenoviral proteins induce autophagy via the upregulation of ATG5 and LC3 [88] or by stimulating BECLIN  1- Bcl-2 dissociation [89, 90]; by contrast, the E4 protein blocks autophagy by acting directly on mTOR signaling. Remarkably, high levels of autophagy in CSCs could represent an useful feature to counteract their resistance to conventional therapy. In 2013, Tong et al. [91] found that an oncolytic adenovirus encoding BECLIN 1 is able to kill LSCs. Interestingly, some OVs have been displayed to regulate autophagy to stimulate both innate and adaptive immune responses, by contributing to antigen presentation and cytokine production during the oncolytic processes. For instance, the autophagy-mediated secretion of ATP in infected-tumor cells activates dendritic cells to produce IL-1β that stimulates IFN-*γ*-dependent T lymphocytes [92]. Overall, OVs represent a great promise for CSC counteraction, and they can be used for both autophagy-inducing and -inhibiting strategies, in order to modulate the CSCs fate.

## Mitophagy in CSCs: a role in metabolic reprogramming

Mitochondria are the organelles responsible for energy production through OXPHOS, synthesis of biomolecules, maintenance of calcium homeostasis, production of reactive oxygen species (ROS), and apoptotic activation [93]. Metabolic reprogramming through the regulation of mitochondrial activities is a key feature of CSCs. It orchestrates their self-renewal capacity, stemness, resistance to toxic agents and also their migration abilities [94–96]. Indeed, it is widely known that many cancer cells rely on aerobic glycolysis, a phenomenon defined as “Warburg effect”, with a prominent decrease in oxidative phosphorylation (OXPHOS) and, therefore mitochondrial functions, even in the presence of functional mitochondria [97, 98]. At variance with this notion, CSCs show a unique metabolic adaptation that is determined by the surrounding environment, such as the hypoxic niche of solid tumors, regions with adequate levels of oxygen (normoxia), active growing regions of the tumor and metastatic sites [94, 95, 99–101]. In a few instances, this wide adaptation has generated controversial results: Vlashi et al. [102] showed that glioma CSCs relied mainly on OXPHOS for energy supply, while other studies described that glioma CSCs are driven by a glycolytic reprogramming, exhibiting more fragmented mitochondria than neuronal stem cells and downregulation of mitochondrial respiratory activity in GSCs [103]. Although CSCs denote an elevated degree of metabolic plasticity, growing evidence suggests that these cells rely more on OXPHOS for energy production, with this being a far more efficient process in ATP generation than glycolysis (as recently reviewed in refs. [94, 95, 101]). In fact, several studies in different tumor types, such as CD133^+ ^cells of glioblastoma and pancreatic ductal adenocarcinoma, LSCs, lung cancer side population cells, and breast cancer, strongly support an OXPHOS phenotype [94, 95, 101].

In fact, in both AML and CML stem/progenitor cells, the inhibition of OXPHOS (obtained by means of inhibitors of complex I or by blocking amino acid metabolism/mitochondrial translation) induces cell death, while in AML it promotes cell differentiation; thus, these data highlight the existing connection between CSCs fate and mitochondrial metabolism [104–107].

**Fig. 3:**
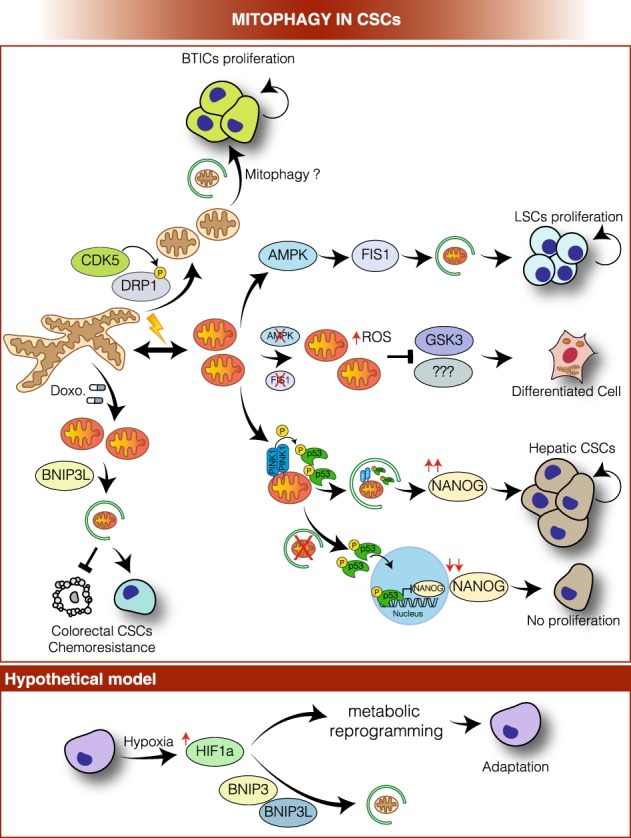
Mitophagy in CSCs, a working model. In brain tumor-initiating cells (BTICs), CDK5-dependent DRP1 activation promotes mitochondrial fission, and (hypothetically) mitophagy, to sustain self-renewal and growth. In leukemia stem cells (LSCs), activated AMPK induces FIS1-mediated mitophagy to guarantee removal of damaged mitochondria, keeping ROS levels under tight control, and, thus, contributing to LSC proliferation. In fact, AMPK or FIS1 loss leads to accumulation of damaged mitochondria and increase of ROS production, which promote GSK3 activation that drives cell cycle arrest and differentiation. Furthermore, in hepatic CSCs, PINK1 on the one hand activates p53 through phosphorylation on the mitochondrial membrane and, on the other hand, mediates mitophagy-dependent p53 degradation, thus favouring NANOG expression and hepatic CSCs proliferation. The suppression of mitophagy efficiency entails the accumulation of activated p53 that translocates to the nucleus, where it inhibits NANOG expression, hindering hepatic CSC proliferation. Moreover, BNIP3L-mediated mitophagy contributes to doxorubicin resistance in colorectal CSCs. A hypothetical model upon hypoxia conditions: hypoxia activates HIF-1α that drives the CSCs metabolic reprogramming. We can hypothesize that HIF-1α endorses BNIP3 and BNIP3L expression and, in turn, these factors mediate mitochondrial degradation and contribute to the switch from oxidative to glycolytic metabolism

Which is thus the role of mitophagy, the selective removal of damaged or superfluous mitochondria by autophagy [108], in such a profound metabolic reprogramming of CSCs (Fig. 3)?

Indeed, mitochondria are highly dynamic structures, undergoing constant fission (regulated by GTPase MFN1,-2 and OPA1) and fusion (regulated by GTPase DRP1 and its accessory factors, including FIS1) to adapt their structure to the energetic and physiological needs of the cell or to ensure their transport or elimination in case of damage [109]. Mitochondrial dynamics also play an important role in regulating mitophagy [110]. It has recently been described that DRP1-driven mitochondrial fragmentation contributes to the acquisition and maintenance of stem cell pluripotency [111, 112] and that this seems to be true also in CSCs [113]. Brain tumor-initiating cells (BTICs), which can be considered a type of neuronal CSCs, boost up mitochondrial fission through CDK5-dependent DRP1 activation to prevent cell death and, thus, to sustain self-renewal and growth [114]. In fact, DRP1 activation in BTICs correlates with poor glioblastoma patient survival [114].

Furthermore, emerging evidence suggests CSC increase antioxidant defense to counterattack ROS production, derived by enhancement of OXPHOS rate; this mechanism assists in the maintenance of their stemness and tumorigenic capacities [94, 101]. To the same aim, CSCs may rely on mitophagy to guarantee the degradation of damaged mitochondria, thus keeping ROS levels under tight control and preventing the activation of programmed cell death [93]. In fact, mitophagy is a potent pro-survival pathway: several mitophagic mechanisms have been identified (reviewed in ref. [115]) and, in some cases, they show some redundancy to ensure efficient and proper elimination of selected mitochondria. Alteration of mitophagy efficiency due, for example, to genetic mutations of key genes, such as PINK1 or PRKN (PARKIN), has been associated with neurological disorders (e.g., Parkinson disease, cancer, heart failure, and aging [116]). Moreover, mitophagy plays a prime role in the maintenance of stem cell pool restoration and homeostasis, and in counteracting senescence by limiting ROS-induced genome damage [117].

One of the most well-characterized mitophagy pathways is represented by the PINK1-PARKIN cascade [118]. PINK1 accumulates specifically on depolarized mitochondria and, through direct phosphorylation, stimulates PARKIN’s E3 ligase activity and recruitment to mitochondria [118]. Therefore, the PINK1-PARKIN system tags damaged mitochondria with ubiquitin to allow recognition by autophagy cargo receptors, such as SQSTM1/p62, optineurin (OPTN), NDP52 and AMBRA1, which, in turn, promote the engulfment of mitochondrion by autophagosomal membrane by binding with LC3/GABARAP family members [115, 118]. Interestingly, loss of PINK1- dependent mitophagy is sufficient to dramatically decrease the efficiency and speed of induced pluripotent stem cell (iPSC) reprogramming from mouse embryonic fibroblasts [119]; this indicates that mitophagy is directly responsible for determining the fate of stem cells. Likewise, in haematopoietic stem cells, mitophagy is fundamental to promote healthy mitochondria degradation and so to maintain quiescence and stemness, mainly to preserve the regenerative capacity of old haematopoietic stem cells [120].

Similarly, LSCs showed a constitutive activation of AMPK, a central regulator of energy and mitochondrial homeostasis that coordinates initiation of autophagy and mitophagy through ULK1 activation [121]. In LSCs, AMPK endorses FIS1-mediated mitophagy to maintain a healthy mitochondrial network and preserve LSC stemness [122]. Intriguingly, inhibition of the AMPK-FIS1 axis leads to a plethora of consistent phenomena, ranging from accumulation of damaged mitochondria, and presumably increased ROS levels, to GSK3 inactivation and cell cycle arrest. All these events drive inhibition of proliferation and induce differentiation of LSCs [122].

Also, Liu et al. [123] discovered that mitophagy positively regulated hepatic CSCs in an unexpected way, by promoting transcriptional activation of NANOG, a key transcription factor known to be required for self-renewal and maintenance of cell stemness [124]. In fact, mitophagy activation fosters autophagosome-mediated p53 sequestration and degradation; PINK directly phosphorylates p53, regulating its localization and consequently its capability to suppress the expression of NANOG [124]. As mentioned above, CSC metabolic adaptation is mainly a result of their surrounding cell environment. Hypoxia is a common condition for those CSCs that are located inside a tumor niche; here, CSCs are able to trigger the switch from OXPHOS to glycolytic metabolism and undergo quiescence [95, 125] in order to increase their fitness and self-renewal potential. Hypoxia leads to activation of both HIF-1*α* and HIF-2*α* that are generally inhibited by VHL upon normoxic conditions [126, 127]. Thus, HIF-1*α* drives CSCs metabolic reprogramming [126] and promotes the expression of several glycolytic proteins inducing in the meantime cell survival [128]. We may also hypothesize that, upon these hypoxic conditions, CSCs may bio-energetically take advantage of BNIP3, BNIP3L/NIX, or FUNDC1-dependent mitophagy, through the activation of HIF-1*α*, in order to guarantee the reduction of mitochondrial mass and avoid activation of apoptosis. In fact, all these three proteins, which act as mitophagy receptors by interacting directly with LC3 through their LIR motif [129], are transcriptionally upregulated by HIF-1*α* during hypoxia [126, 130]. A recent study reports that in the context of somatic cell reprogramming, a BNIP3L-dependent mitophagy-mediated metabolic shift toward glycolysis is essential [131], indicating that BNIP3L, but also BNIP3 and FUNDC1, require further investigation in order to elucidate their role in regulating CSCs fate.

So far, we have discussed mitophagy as a *positive* mechanism that sustains CSCs in adverse conditions or during their metabolic shift. However, an excessive rate of mitophagy flux can also confer chemoresistance: CD133^+ ^/CD44^+ ^CSCs from HCT8 human colorectal cancer cells CSCs have been shown to use BNIP3L-mediated mitophagy to escape from doxorubicin-induced cell death [132].

## Autophagy, CSCs, and clinical implications

In general, it is well defined that autophagy and mitophagy could represent a promising target for counteracting CSCs aggressiveness. However, it is important to remind that chloroquine and its derivatives (mainly hydroxychloroquine) are used in many clinical trials, often in combination with conventional anticancer treatments [133].

Further and of the highest importance, CSC heterogeneity and patient-specificity makes the situation more complex than previously thought. We are still far away from dealing with useful tools to set up novel drug combinations allowing us to eradicate CSCs or at least to inhibit their proliferation. One of the main ambitions of the actual worldwide efforts is to integrate latest discoveries into innovative treatment strategies. Recent experimental observations suggest that autophagy inhibition and autophagy activation may both be used as encouraging approaches for sensitizing CSCs to therapy. In light of the findings described above, the efficacy of chloroquine application in anti-CSCs therapy could depend on the tumor type that is being treated and autophagy-dependency of CSCs might also play a role in this context. Nowadays much more specific and potent lysosome inhibitors than chloroquine are being established, such as Concanamycin A, a selective inhibitor of V-ATPase that prevents lysosome and endosome acidification, or E64d, an inhibitor of cathepsins B, H, and L, or pepstatin A, inhibitor of cathepsins D and E [134]; these drugs offer the opportunity to develop a wide combinations of different therapies. However, the prevention of autophagosome degradation does not affect autophagosome formation and cargo sequestration. As mentioned above, mitophagy mediates the removal of damaged mitochondria preventing oxidative stress and activation of apoptotic cell death [135]; indeed, lysosomal inhibitors may not be able to reduce the rate of mitochondrial sequestration by autophagosomes, and this could limit the drug efficacy on CSC effects that rely on mitophagy. Thus, in this context, the use of drugs against the initial phases of autophagy, such as VPS34 (i.e., SAR405 [136] or PIK-III [137]) or ULK1 (i.e., MRT68921 [138]) inhibitor, may provide better results.

Moreover, recent findings prompt us to hypothesize new and intriguing scenarios based on the regulation of autophagy in the microenvironment surrounding CSCs. For example, malignant tumor cells induce autophagy in the microenvironment and distal tissues to support their own growth by increasing the availability of recycled nutrients. Autophagy inhibition within the tumor causes a moderate effect on tumor progression, while autophagy inhibition through oral administration of chloroquine leads to a more noticeable reduction in tumor growth and invasion [139]. There is a metabolic crosstalk between CSCs, non-CSCs and cancer- associated fibroblasts (CAFs) that generate metabolic symbiosis [140]; thus, we can hypothesize that targeting non-CSCs and CAFs with autophagic inhibitors may lead to a reduction of nutrient availability and this, in turn, may negatively impact on CSC innate resistance mechanisms against chemotherapy. However, interventions that inhibit autophagy could have unintended side effects on anticancer immune surveillance; in recent years, it turned out that fasting- and caloric-restriction-dependent autophagy induction improve anticancer immune surveillance, hence promoting tumor growth arrest and improvement of the chemotherapeutic outcome [141].

Consequently, getting insights into the regulatory factors and the molecular mechanisms by which autophagy exploits its function in CSCs is fundamental for developing more effective and safe antitumor strategies. Besides the use of autophagy inhibitors/activators in combination with chemotherapeutic drugs, the investigation of autophagy in both immune and virotherapy could be crucial to identify new strategies against CSCs that escapes conventional therapy. In addition, it is central to remind that solid tumors usually grow in low oxygen environments, which create a niche to protect CSCs, and make them more aggressive and resistant to cell death. Further investigations are thus necessary to dive into the role of autophagy in the crosstalk between stromal cells, endothelial cells, and tumor-infiltrating innate and adaptive immune cells. Importantly, these cells may have divergent necessities for autophagy that could make difficult to conceive autophagy-targeting therapy.

Of note, more work is also necessary for developing new and reliable methods for quantifying autophagy flux in patient samples. Certainly, the isolation of CSCs from the blood of the patients may represent a powerful way for monitoring basal autophagy. Moreover, taking advantage from the RNA sequencing approach, a revolutionary tool for transcriptome analysis, it could be possible to predict the state of autophagy activation based on the expression profile of these cells.
